# Development of Sustainable Red Algae–Sisal Fiber Composite Films via Doctor Blading

**DOI:** 10.3390/polym18030424

**Published:** 2026-02-06

**Authors:** Matthew Richards, Joshua Baird, Noah Serda, Vuong Do, Yanika Schneider

**Affiliations:** Department of Chemical and Materials Engineering, San Jose State University, One Washington Square, San Jose, CA 95192, USA

**Keywords:** red algae polymer, sisal fibers, biocomposite, thermal analysis, mechanical analysis, kappa-carrageenan, doctor blading

## Abstract

This study investigated the properties of red algae (RA) biocomposite films reinforced with natural sisal fibers and plasticized with glycerol. The polymer was extracted from locally sourced red seaweed and combined sisal fibers at varying fiber loadings (0–45 wt%) using the doctor blading technique. Composite films were analyzed using a variety of methods to evaluate the chemical composition, thermal behavior and mechanical performance. Infrared spectroscopy confirmed the presence of kappa-carrageenan as the dominant polysaccharide in the RA matrix, whereas elemental analysis verified the dilution of sulfur content and enrichment of carbon with increasing fiber incorporation. Thermal stability increased with fiber loading, peaking at 30 wt% sisal fiber before decreasing slightly at 45 wt% due to poor fiber dispersion. Mechanical testing demonstrated an optimal balance between strength and flexibility at 30 wt% sisal fiber, with a 37% increase in strength compared to the pure RA film. Overall, the findings demonstrate that sisal fiber reinforcement enhances the structural integrity and stability of RA-based films, supporting their potential as biodegradable alternatives to petroleum-based plastics.

## 1. Introduction

Developing effective and sustainable alternatives to traditional petroleum plastics is one of the primary problems faced by the scientists and engineers of this generation. In recent years, researchers have investigated red algae (RA) polysaccharides in their search for sustainable alternatives to petroleum-based materials. These biopolymers have the advantage of being renewable and carbon negative with a simple, eco-friendly extraction process due to their water solubility [1]. RA encompasses thousands of species, most of which are marine, that can be readily found in oceans all over the world. Their cell walls and intercellular matrices are often composed of unique polysaccharides called sulfated galactans. One such sulfated galactan is carrageenan, a common food additive utilized for stabilization and gelling [2]. Unlike brown algae-derived biopolymers, the polymers in RA can be extracted without the need for any pH treatment [3], leading to gentler processing conditions for biopolymer creation and increasing the range of applications. For example, researchers have used RA in various fields such as nanotechnology, biomedical coatings, battery applications, adhesives and green concrete [3,4,5,6,7,8,9,10,11,12,13,14,15,16,17,18,19,20,21,22,23,24].

Despite its promising applications, the mechanical and thermal properties of RA materials are generally inferior to those of petroleum-based plastics [25]. Dang et al. recently provided a comprehensive overview of different algae-based materials [1]. The authors demonstrated that polysaccharide properties are affected by the amount of glycolic bonds, the degree of branching and functional groups. Overall, RA polymers were found to exhibit brittle tensile properties due to strong hydrogen bonding.

A common way to improve the elasticity of brittle polymers is by incorporating plasticizers. These molecules increase the space between chains, leading to enhanced material ductility, which also tends to reduce mechanical strength. Common plasticizers include glycerol, polypropylene glycol and fructose [1]. Recently, Jang et al. compared the effect of several different plasticizers on RA films produced by solution casting [3]. The authors determined that fructose acted as the best plasticizer, with glycerol also providing satisfactory results. The authors also incorporated nanoclays, which increased mechanical properties relative to pure films, offsetting the mechanical loss due to plasticization.

Reinforcements such as mineral fillers or fibers are typically added to increase mechanical properties of materials. Chopped natural fibers such as sisal [26], kenaf [27] and chitin whiskers [28] have been added to various polymer matrices to enhance strength and thermal properties. Sisal is a lignocellulosic fiber mainly composed of cellulose with smaller fractions of hemicellulose and lignin. Sisal fibers are favored due to their good mechanical properties and low water absorption. Gudayu et al. used short sisal fibers as reinforcement in PET thermoplastic injection-molded parts [26]. At 40 wt% sisal fiber loading, the tensile modulus of the PET increased by 137%. Accordingly, when used in conjunction with a plasticizer, fiber reinforcements can create a strong yet flexible composite material from an otherwise weak and brittle biopolymer.

Although polysaccharides from red algae have been extensively researched as eco-friendly alternatives, previous attempts to optimize their mechanical properties have been mostly limited to plasticization and nano-filler reinforcement [13,19,28,29]. The use of natural fiber reinforcement has been investigated extensively in thermoplastics as well as in alginate systems, but there have been no reports of its use in carrageenan matrices [26]. Moreover, the preparation of seaweed-derived films has been mostly limited to solution casting. Doctor blading offers clear scalability advantages for thin-film preparation, but seldom has been applied to biopolymers [30]. The aim of our research is to prepare a novel biocomposites using naturally processed κ-carrageenan and glycerol reinforced with chopped natural sisal fibers via the doctor blade technique. Following film fabrication, the biocomposites will be evaluated using chemical, thermal, and mechanical characterization methods to examine structure–property relationships and assess their performance as a sustainable alternative to petroleum-based plastics.

## 2. Materials and Methods

RA was collected from a beach in Monterey, CA, USA. Sisal fibers were purchased from a local craft store (Santa Clara, CA, USA) and glycerol was obtained from Fisher Chemical (Pittsburgh, CA, USA).

### 2.1. RA Polymer Extraction

**Figure 1:**
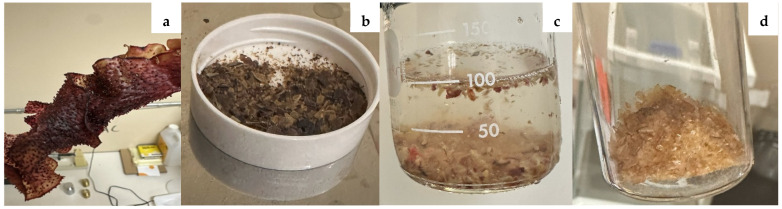
RA solution preparation method. Starting from RA raw seaweed (**a**), followed by grinding (**b**) and extracting (**c**) the polymer from RA seaweed in water. The final drying and grinding stages produced a solid polymer powder (**d**) used in subsequent film fabrication.

Figure 1 shows the RA preparation method. First, RA was washed with DI water to remove any large debris. The polymer was then dried at 60 °C for 30 min and ground-up using a Mr. Coffee bullet blender to produce a coarse powder. Approximately 2 g of RA powder was added to ~125 mL of DI water, and the mixture was stirred on a hot plate at 50–60 °C for 15 min. Afterwards, the heat was turned off, and the mixture was allowed to continue stirring overnight. The resulting solution was then filtered through a fine Nylon mesh bag to remove large particles and centrifuged at 5000 rpm for 10 min using a LAB Fish LC-8S centrifuge (JOANLAB, Huzhou City, China). The resulting supernatant was placed in a large ceramic mortar bowl and evaporated to dryness for approximately 12 h overnight at 60 °C in a Standard Environmental Systems EB/3 evaporating oven. The resulting RA solids (~1 g or 50% yield) were then ground up into a fine powder and stored for later use.

### 2.2. Sisal Fiber Preparation

**Figure 2:**
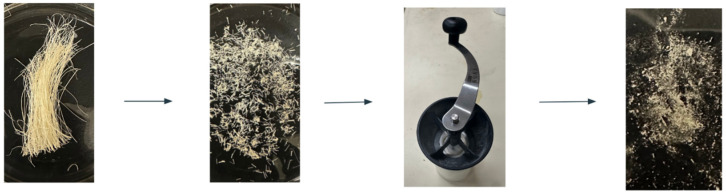
The sisal fiber preparation method involved cutting raw fibers into short segments and then grinding them to a fine powder. This process improved the fiber dispersion in the RA–glycerol matrix.

Sisal fibers were prepared by first cutting the fibers with scissors into small pieces. Next, they were repeatedly processed using a KONA manual ceramic burr coffee grinder (IDYLC Homes, Wilmington, DE, USA) until they were a powder consistency, as shown in Figure 2.

### 2.3. Composite Precursor Preparation

The dried RA polymer was dissolved in DI water at 40–50 °C to generate a 10 wt% solution. Once the polymer was fully dissolved, glycerol was added equal to the weight of the RA polymer in solution. This mixture was then stirred at 40–50 °C for approximately 15 min to ensure full homogenization. The precursor solution was then allowed to remain undisturbed such that any bubbles created from the mixing process could rise to the top of the mixture. Finally, the processed sisal fibers were added to the precursor and stirred gently to evenly distribute fibers without creating air bubbles. The weight fraction of the fibers was calculated with respect to the dry weight of the RA polymer in solution.

### 2.4. Doctor Blading Film Preparation

**Figure 3:**
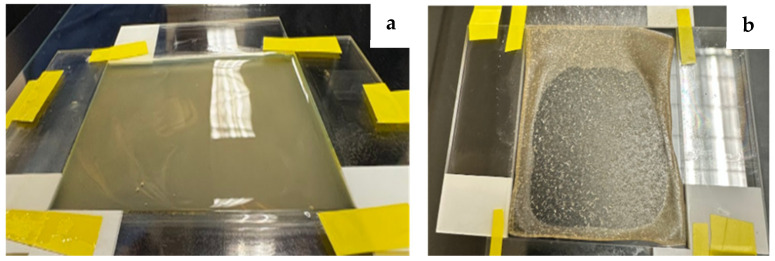
Photographs show film formation using the doctor-blading technique. Image (**a**) shows the wet precursor layer is spread within a fixed-height glass mold. The dried film (**b**) reveals a reduction in thickness and uniform film appearance after solvent evaporation.

Films were generated via doctor blading technique using a 20 × 20 cm glass pane as a base and 7.5 × 2.5 × 0.1 cm glass slides stacked two deep. The pieces of glass were arranged to create a rectangular enclosure approximately 7.6 × 6 × 0.2 mm (see Figure 3). The precursor was then applied to the mold by pouring the solution onto one edge of the rectangle and using the edge of an additional slide to evenly spread the composite mixture across the entire surface of the enclosure. The cast precursor was then left in a fume hood to dry overnight. Finally, the resulting dried film was removed from the mold and further dried in an Econotherm laboratory oven (Thermo Fisher Scientific, Waltham, MA, USA) at approximately 60 °C for 30–60 min to remove any excess moisture.

### 2.5. Film Characterization

#### 2.5.1. Chemical Characterization

The surface of each film (N = 1) was examined in attenuated total reflection (ATR) mode using a Thermo-Nicolet iS50 Fourier Transform Infrared (FTIR) spectrometer. Approximately 12–20 scans were performed with a resolution of 4 cm^−1^. OMNIC 9.12 software was used to perform data analysis.

XRF was performed on films (N = 3) using a Rigaku Primus II WDXRF (Rigaku Corporation, Tokyo Japan) with a Rhodium x-ray tube source. The films were cut to a diameter of 20 mm, placed into sample holders and analyzed directly under a vacuum atmosphere. Quantification was performed using the Fundamental Parameters standardless quantification process with the Rigaku ZSX Version 7.99 software. The fundamental parameters approach uses x-ray physics coupled with established sensitivity factors for pure elements. Relative accuracy by this method usually ranges from better than 5% up to ~20% for major elements.

#### 2.5.2. Thermal Characterization

Thermogravimetric analysis (TGA) was performed on films (N = 2) using a PerkinElmer TGA 800 (PerkinElmer, Shelton, CT, USA) on ~5–10 mg of sample. Samples were heated in ceramic pans at 10 °C/min from 30 °C to 600 °C under N_2_ flow, followed by air at 10 °C/min until 800 °C. Trios Software was used for data processing. Differential scanning calorimetry (DSC) analysis was performed on films (N = 2) using a TA Instruments Q20 (TA Instruments, New Castle, DE, USA) on 10–20 mg of sample. Samples were heated in aluminum pans at 10 °C/min from 30 °C to 200 °C under a N_2_ flow.

#### 2.5.3. Mechanical Characterization

Tensile testing on films (N = 4) was conducted on an Electropuls E1000 Instron machine (Norwood, MA, USA) following the ASTM D882 standard for tensile testing of thin films less than 1.0 mm in thickness [31]. The tests utilized a 1 kN load cell at a strain rate of 2.5 mm/min. The samples were cut to 5 cm in length, 1 cm in width, and approximately 0.1–0.4 mm in thickness. The average gauge lengths for the 0, 15, 30 and 45 wt% samples were 2.23, 2.21, 2.28 and 2.28 cm, respectively. Additional details are provided in the Supplementary Materials (Table S1).

#### 2.5.4. Morphological Characterization

Scanning Electron Microscopy (SEM) was performed to study the morphology of the RA composite films using FEI Quanta 200 (FEI, Hillsboro, OR, USA). Prior to SEM imaging, samples were coated with a thin conductive layer of gold using a Denton IV sputter coater (Denton Vacuum, Moorestown, NJ, USA), employing argon plasma as the sputtering gas.

## 3. Results

### 3.1. Film Formation

**Figure 4:**
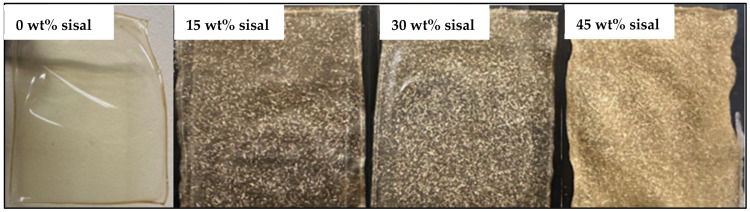
Photographs of RA composite films containing 0–45 wt% sisal fibers.

RA films were created using an aqueous 10 wt% stock solution of equal parts RA and glycerol with varying concentrations of sisal fibers. Figure 4 presents films containing 0–45 wt% sisal fiber, showing a progression from transparent to increasingly opaque.

### 3.2. Chemical Characterization Results

#### 3.2.1. ATR-FTIR Results

**Figure 5:**
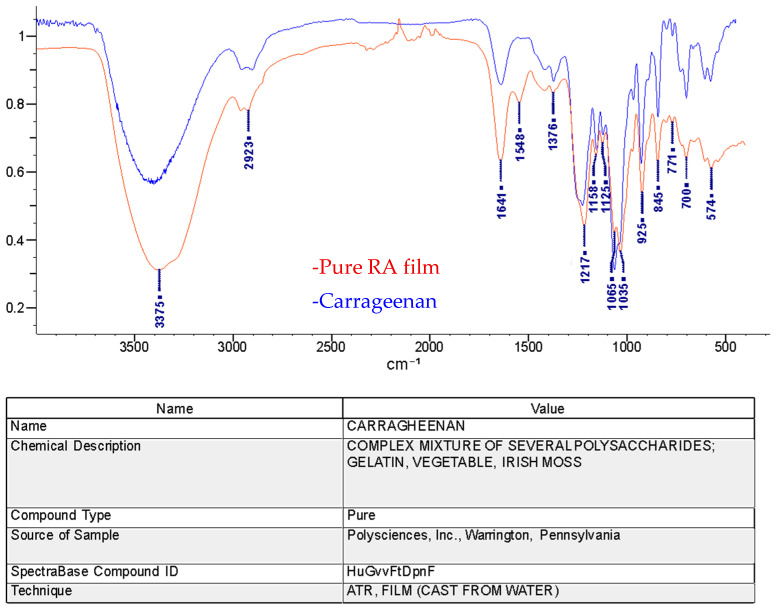
ATR-FTIR Spectra of a pure RA film and a reference kappa-carrageenan sample.

ATR-FTIR was used to identify the molecular structure and determine the type of polysaccharide present in the RA film. Figure 5 compares the FTIR spectrum of the pure RA film with a reference carrageenan spectrum from the Wiley BioRad library. The similarity between the two FTIR spectra confirms that the primary polysaccharide in the film is carrageenan. The presence of peaks at 925 and 845 cm^−1^ indicates that this material is likely kappa-carrageenan [32]. The FTIR results also suggest that the extraction procedure preserved the sulfate ester group, as evidenced by the absorption peaks at 1210 and 1030–1070 cm^−1^ in Figure 5 [33,34]. The presence of a carrageenan backbone is consistent with the high content of sulfur and propensity for water absorption discussed later [35]. Finally, the intensity of the peak at 1641 cm^−1^ coupled with the peak at 1548 cm^−1^ suggests that polyamides or residual proteins were also detected in the extracted RA biopolymer [34].

**Figure 6:**
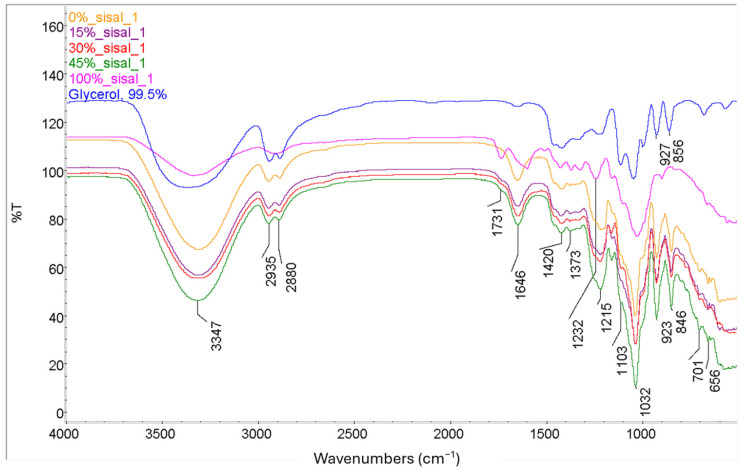
FTIR spectra of glycerol, sisal and plasticized biocomposite films.

Figure 6 compares the composite films with the individual components. Individual FTIR spectra are provided in Supplementary Materials (Figures S1–S5). The results indicate that all films contain glycerol-related absorbances, including the CH peaks at 2935 and 2880 cm^−1^ and the OH peaks 927 and 856 cm^−1^. The incorporation of sisal fibers resulted in minimal changes from the pure RA film. The sisal fibers utilized in this study contain an ester peak at 1731 cm^−1^, which can be weakly observed in the composites but not in the pure RA film. In contrast, the sharp lignin peak at 1596 cm^−1^ is not detected in the composite films (see Figure S6). Table 1 provides the FTIR peak assignment for the vibrations observed in the plasticized films.

#### 3.2.2. XRF Results

**Figure 7:**
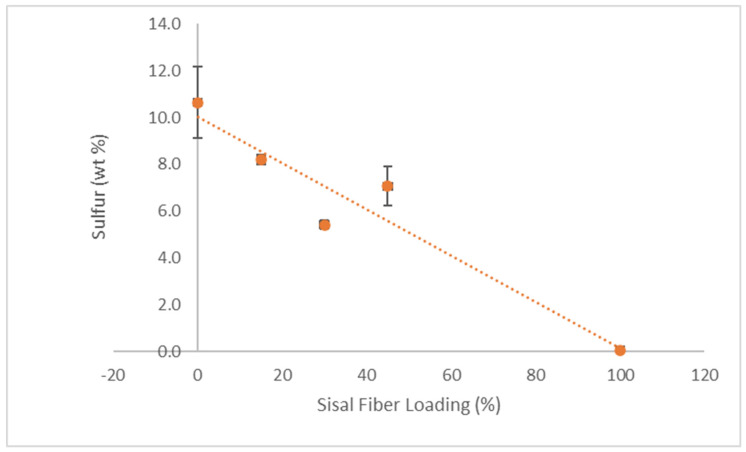
Sulfur content (by XRF) of RA-sisal composite films as a function of fiber loading (N = 3).

XRF analysis was conducted to determine the elemental composition of the RA and composite films, and the results are shown in Table 2. Sisal fibers contained high levels of oxygen (61.5 wt%) and carbon (36.8 wt%) due to their high cellulose content. The control RA film (0% sisal) also contained high oxygen content (64.5 wt%), but far less carbon (12.8 wt%) than sisal. Moreover, a much higher sulfur concentration (10.6 wt%) was detected in the pure RA film compared to sisal fiber (0.045 wt%). High sulfur content is characteristic of sulfated polysaccharides such as carrageenan. Figure 7 shows sulfur content decreasing with increasing fiber concentration, consistent with the dilution of the sulfated carrageenan phase. Note that the 45 wt% film had a higher sulfur content than expected, likely due to inhomogeneity. These results suggest that sulfur and carbon content may provide a benchmark by which to measure fiber mixing within the sulfated carrageenan/glycerol matrix. Composite films also contained minor elements such as potassium, sodium, calcium, chlorine, and magnesium, which may be due to their marine origin.

### 3.3. Thermal and Mechanical Analysis Results

#### 3.3.1. Thermogravimetric Analysis Results

**Figure 8:**
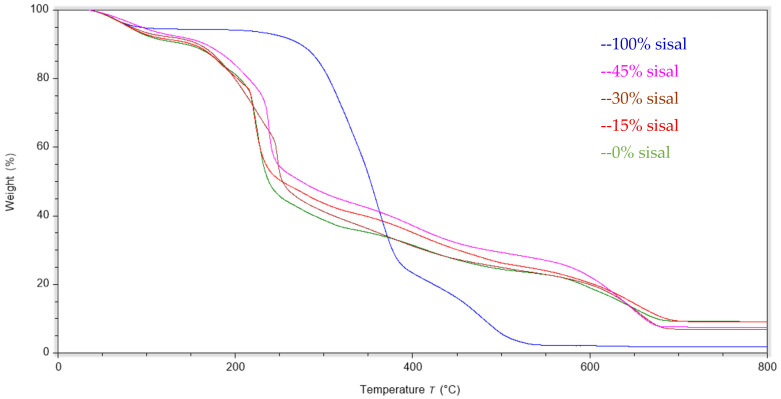
TGA thermograms of RA-sisal composite films with varying sisal content.

TGA analysis was performed on the biocomposite films, and the results are shown in Table 3 and Figure 8. Additional details are provided in the Supplementary Materials (see Figures S7–S11). The volatile content was calculated from the initial mass loss occurring between 40 °C and 120 °C, corresponding primarily to physically absorbed and weakly bound moisture in the films. Increasing fiber concentration resulted in a decrease in the volatile content of the films up to 30 wt% sisal fiber, reaching a steady state of 8.2–8.4 wt%. The moisture content of the sisal fiber was more than 2-fold lower than that of the pure RA film.

For all polymer films, the most significant mass loss event occurred in the temperature range of approximately 200 to 300 °C, corresponding to the decomposition of the glycerol plasticizer and functional groups on the RA polymer chain. The amount of weight loss during this step is noted in Table 3 in the mass loss column. The mass loss decreases from 35 wt% for pure RA film to 24 wt% for the 45 wt% composite, suggesting that it is related to the relative glycerol concentration in the film. Although the boiling point of glycerol is approximately 290 °C, it has been shown in the literature that the incorporation of only 20 wt% water decreases the boiling point to 121 °C [36].

Moreover, thermal stability of the films can be assessed by comparing the T_max_ temperatures of this major transition. At low fiber loadings, the T_max_ slightly decreased from 227 °C for the pure RA film to 221 °C for the 15 wt% sisal composite film. However, the 30 wt% composite film showed an increase in the T_max_ by more than 20 °C to 248 °C. At highest fiber loading of 45 wt%, the T_max_ value decreases slightly (239 °C), remaining significantly higher than that of the pure RA film.

Another transition is observed at ~400 °C in the composite films (see Figures S7–S11), accounting for approximately ~10% mass loss. This transition is due to the degradation of the polysaccharide backbone and char formation [24,35]. Moreover, the residue weight in Table 3 corresponds to the composite weight remaining after heating to 800 °C. The pure RA film has the highest residue value of 9.2 wt%, which decreases with increasing fiber loading to 7 wt% for the 30 wt% composite. These results suggest that the high sulfur content of the RA polymer is likely contributing to the elevated residue weight by producing charring species at elevated temperatures [37]. This effect becomes diluted with the increasing incorporation of sisal fibers, which only generate ~1.9 wt% char.

Overall, the TGA results confirm that proper fiber dispersion is critical for realizing an optimum balance between mechanical and thermal properties. The sisal fiber reinforces the polymer matrix, decreasing the susceptibility of the polymer chains to thermal degradation.

#### 3.3.2. Differential Scanning Calorimetry (DSC) Results

**Figure 9:**
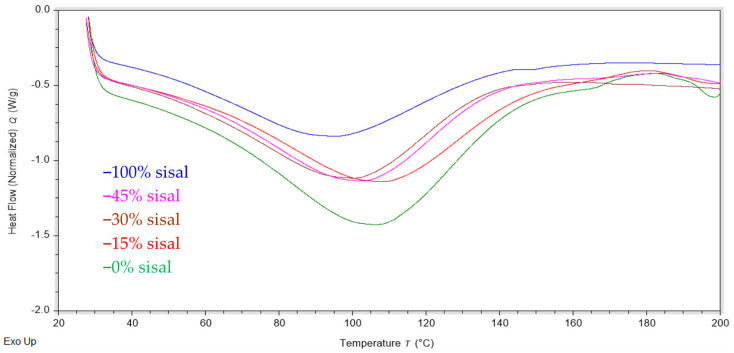
DSC heating curves of RA-sisal composite films with varying fiber content.

DSC thermal analysis was conducted on the biocomposites, and the results are summarized in Table 3 and Figure 9. DSC analysis revealed that the heat flow corresponding to the water absorption decreased with increasing fiber loadings. In fact, the 45% fiber sample value had the lowest water absorption value of the composites at 199 J/g compared to 292 J/g for pure RA film. Consistent with the TGA results, the introduction of sisal fiber decreased hydrogen bonding, leading to reduced water uptake [38,39]. Specifically, rigid sisal fibers disrupt the hydrogen-bonding network formed between water, glycerol and carboxylate functionalities in the RA films. Note that the FTIR spectra of all biocomposites show a broad O–H stretch at 3347 cm^−1^, but no significant peak shifts with increasing fiber (Figure 6). This result indicates that the nature of hydrogen-bonding sites does not change due to sisal incorporation. Thus, the lower water uptake is likely due to the fibers physically replacing some of the more water-absorbing carrageenan/glycerol sites, and not due to the formation of new chemical bonds.

#### 3.3.3. Mechanical Testing Results

**Figure 10:**
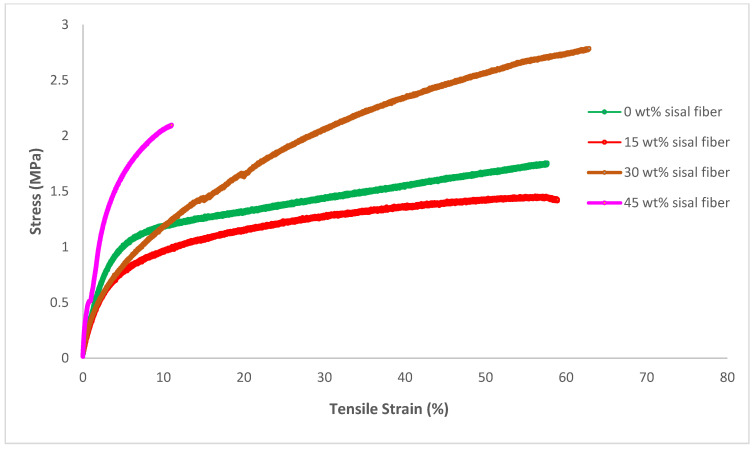
Representative stress–strain curves for RA-sisal composite films with varying fiber loading.

Tensile testing was conducted to determine the effect of varying sisal fiber content on the mechanical properties, and the results are summarized in Table 4 and Figure 10. Attempts to generate dog bones from the pure RA film were not successful due to the brittleness of the biopolymer. The plasticized RA film exhibited an ultimate tensile strength of 1.83 MPa and a strain at break of 67%. In contrast, the strongest composite (30 wt% fiber) increased the strength by more than 37% to 2.51 MPa, while maintaining a similar elongation at break. This result indicates that this biocomposite exhibits significantly higher toughness compared to the pure polymer. The incorporation of sisal fibers initially slightly decreased the Young’s modulus from 8.62 to 8.10 MPa for the 15 wt% sisal fiber composite. Increasing the sisal content to 30 wt% resulted in a slight increase in the modulus to 9.03 MPa. Finally, the 45 wt% composite showed an almost 5-fold increase in modulus to 41 MPa. However, the highest fiber loading exhibited a significant reduction in the elongation at break and a small decrease in strength. This embrittlement may be due to poor dispersion of the fibers at high fiber concentration. Overall, these results confirm the reinforcing effect of sisal fiber, agreeing with previous studies [40,41].

#### 3.3.4. Morphological Evaluation Results

**Figure 11:**
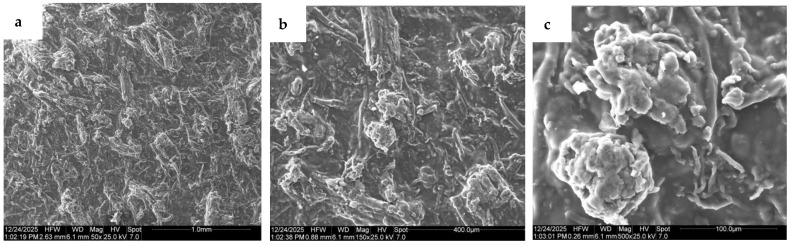
SEM images of 45% sisal fiber composite film at (**a**) 50×, (**b**) 150× and (**c**) 500× magnification.

SEM analysis was performed on the composite films, and the results for the 45% sisal composite are shown in Figure 11. Supporting Information contains additional images of the pure RA (Figure S12) and composite films (Figures S13–S15). The RA film was not smooth with areas of higher roughness and crystalline features. The addition of fibers gradually changed the morphology, resulting in a more homogeneous surface without the crystalline features. The fibers appeared to be fully coated with the RA polymer and well distributed in the polymer matrix. Even at the highest fiber loading, there is no evidence of uncoated sisal fibers. That said, at 500× magnification, the agglomeration of small fibers can be observed (see Figure 11c). These clumps of fibers act as stress concentrators and may explain the poor mechanical properties observed at the highest fiber loading.

## 4. Discussion

This study successfully investigated the effect of sisal fiber on RA composites. Composite films were created using a series of simple processes: polymer extraction, plasticization, fiber incorporation, and film preparation utilizing the doctor-blading method. Compared to solution casting, this process was much simpler and resulted in uniform films with up to 30% sisal fiber loading.

XRF characterization of the pure film revealed high sulfur content, consistent with the kappa-carrageenan species detected by FTIR. As fiber loading increased, sulfur content decreased, confirming successful incorporation of the sisal fiber into the biopolymer matrix. Compared to the pure RA film, the sisal biocomposites showed enhanced mechanical and thermal properties. DSC and TGA revealed that increasing fiber content led to decreased water retention, ultimately resulting in reduced volatiles and enthalpy of water desorption. At very high fiber content (45 wt% sisal), poor dispersion was accompanied by lower mechanical and thermal properties. SEM revealed the agglomeration of small fibers into clumps, which may act as stress concentrators and thereby decrease the elongation at break of the biocomposite.

Accordingly, the optimal fiber loading for this system was 30% sisal fiber, which showed a 40% increase in strength and modulus, while maintaining the same elongation at break. Moreover, the maximum degradation temperature was 20 °C higher than that of the pure RA film. Overall, these findings are promising and warrant further investigation. For example, these composites can be further optimized by applying a coating onto the fibers to enhance the matrix-fiber interface or utilizing ultrasonic mixing to enhance compatibilization [42,43,44]. Similarly, other plasticizers and different loadings can be investigated to improve mechanical properties.

## 5. Conclusions

This study demonstrated that κ-carrageenan biocomposites plasticized with a glycerol and reinforced with chopped sisal fibers can be produced efficiently using the doctor-blading technique. Chemical analysis revealed that the incorporation of fibers does not affect the structure of the κ-carrageenan, while element analysis confirmed that the sulfated network is diluted with increased fiber content. More importantly, sisal fibers decreased moisture absorption, increased thermal stability and improved mechanical properties. Although this is the first study on RA-sisal biocopmposites and this system requires significant optimization, the promising results together with the simple and scalable method highlight the viability of these materials as alternatives to petroleum-based plastics.

## Figures and Tables

**Table 1:** Functional Groups in RA Biocomposite Films.

FTIR Vibration (cm^−1^)	Functional Group
3347	O-H Stretch
2935 and 2880	C-H Stretch
1731	Ester C=O Stretch
1646	O-H Bending and C=O Amide I Stretch
1215	S=O Asymmetric Stretch
1157	C-O Stretch
1103 and 1032	C-O Stretch
923	C-C Stretch
846	C-C Bending

**Table 2:** Compositional Analysis of RA Biocomposite films (N = 3) ^a^.

	0 (wt%)	15 (wt%)	30 (wt%)	45 (wt%)	100 (wt%)
C	12.8 ± 2.5	19.5 ± 0.5	24.8 ± 0.5	22.5 ± 0.5	36.8 ± 0.62
O	64.5 ± 1.5	63.7 ± 0.5	63.8 ± 0.8	63.0 ± 1.1	61.5 ± 0.6
Na	1.89 ± 0.19	2.14 ± 0.03	1.80 ± 0.04	2.04 ± 0.04	0.053 ± 0.06
Mg	0.71 ± 0.05	0.85 ± 0.06	0.75 ± 0.05	0.80 ± 0.07	0.13 ± 0.02
Al	0.069 ± 0.038	0.032 ± 0.004	0.055 ± 0.005	0.062 ± 0.013	0.029 ± 0.018
Si	0.10 ± 0.05	0.048 ± 0.007	0.062 ± 0.006	0.083 ± 0.020	0.040 ± 0.027
P	0.12 ± 0.01	0.065 ± 0.005	0.087 ± 0.002	0.055 ± 0.002	0.034 ± 0.004
S	10.6 ± 1.5	8.45 ± 0.44	5.41 ± 0.18	7.06 ± 0.83	0.045 ± 0.005
Cl	0.41 ± 0.11	0.30 ± 0.011	0.22 ± 0.01	0.28 ± 0.01	0.036 ± 0.031
K	6.88 ± 0.96	4.04 ± 0.19	2.38 ± 0.08	3.31 ± 0.25	0.60 ± 0.04
Ca	1.84 ± 0.31	0.92 ± 0.11	0.63 ± 0.02	0.76 ± 0.14	0.76 ± 0.01

^a^ Trace elements were also detected such as Br, Sr and metals.

**Table 3:** Thermal Properties of RA-sisal composite films ^a^.

Fiber %	Volatiles (wt%)	Mass Loss (wt%)	T_max_ (°C)	Residue (wt%)	Desorption Enthalpy (J/g)	Desorption Temp (°C)
0	9.5 ± 1.5	34.6 ± 1.7	226.6 ± 1.2	9.2 ± 0.1	292 ± 31	104.5 ± 3.1
15	9.2 ± 2.1	30.1 ± 1.5	221.1 ± 0.5	9.1 ± 0.04	233 ± 16	111.7 ± 4.9
30	8.2 ± 1.2	20.1 ± 3.2	247.8 ± 0.1	7.0 ± 0.2	219 ± 19	106.0 ± 8.9
45	8.4 ± 0.3	24.3 ± 0.5	238.5 ± 1.1	7.5 ± 0.1	199 ± 5	104.7 ± 3.0
100	4.1 ± 2.3	71.8 ± 1.7	362.5 ± 3.2	1.9 ± 0.04	153 ± 7	96.3 ± 1.6

^a^ Volatiles, Mass Loss, T_max_ and Residue are from TGA analyses; the Desorption Enthalpy and Desorption Temperature are from DSC.

**Table 4:** Mechanical Properties of RA-sisal composite films (N = 4).

Fiber wt%	Young’s Modulus (MPa)	Ultimate Tensile Strength (MPa)	Elongation at Break (%)
0	8.62 ± 1.39	1.83 ± 0.06	67 ± 6
15	8.01 ± 1.26	1.25 ± 0.19	69 ± 9
30	9.03 ± 0.21	2.51 ± 0.23	65 ± 10
45	41.0 ± 6.98	2.19 ± 0.22	18 ± 6

## Data Availability

The original contributions presented in this study are included in the article. Further inquiries can be directed to the corresponding author.

## Supplementary Materials

The following supporting information can be downloaded at https://www.mdpi.com/article/10.3390/polym18030424/s1, Table S1: Mechanical testing specimen dimensions; Figure S1: FTIR spectrum of 0% sisal fiber sample; Figure S2: FTIR spectrum of 15 wt% sisal sample; Figure S3: FTIR spectrum of 30% sisal fiber sample; Figure S4: FTIR spectrum of 45% sisal fiber sample; Figure S5: FTIR spectrum of 100% sisal fiber sample; Figure S6: Comparison of 0, 45 and 100% sisal fiber samples; Figure S7: TGA Thermogram and derivative plot of the 0% sisal fiber sample; Figure S8: TGA Thermogram and derivative plot of the 15% sisal fiber sample; Figure S9: TGA Thermogram and derivative plot of the 30% sisal fiber sample; Figure S10: Thermogram and derivative plot of the 45% sisal fiber sample; Figure S11: TGA Thermogram and derivative plot of the 100% sisal fiber sample; Figure S12: SEM images of RA film (a) 50× and (b) 150×; Figure S13: SEM images of 15% sisal film (a) 50× and (b) 150×; Figure S14: SEM images of 30% sisal film (a) 50× and (b) 150×; Figure S15: SEM images of 45% sisal film (a) 50× and (b) 150×.
