# Supplementary material for: Development of Sustainable Red Algae–Sisal Fiber Composite Films via Doctor Blading
Source: Polymers (Basel). 2026 Feb 6;18(3):424. doi: 10.3390/polym18030424 (PMC12899586; doi:10.3390/polym18030424)
Supplement: Supplementary file 1 [file polymers-18-00424-s001.zip › polymers-4050442-supplementary.pdf]

SUPPLEMENTAL MATERIAL

Article

Development of Sustainable Red Algae–Sisal Fiber Composite Films via Doctor Blading

Matthew Richards, Joshua Baird, Noah Serda, Vuong Do and Yanika Schneider *

Department of Chemical and Materials Engineering, San Jose State University, One Washington Square, San Jose, CA, 95192

* Correspondence: yanika.schneider@sjsu.edu; Tel.: +1-408-759-3009

This document contains additional FTIR, TGA and SEM data along with mechanical testing specimen information.

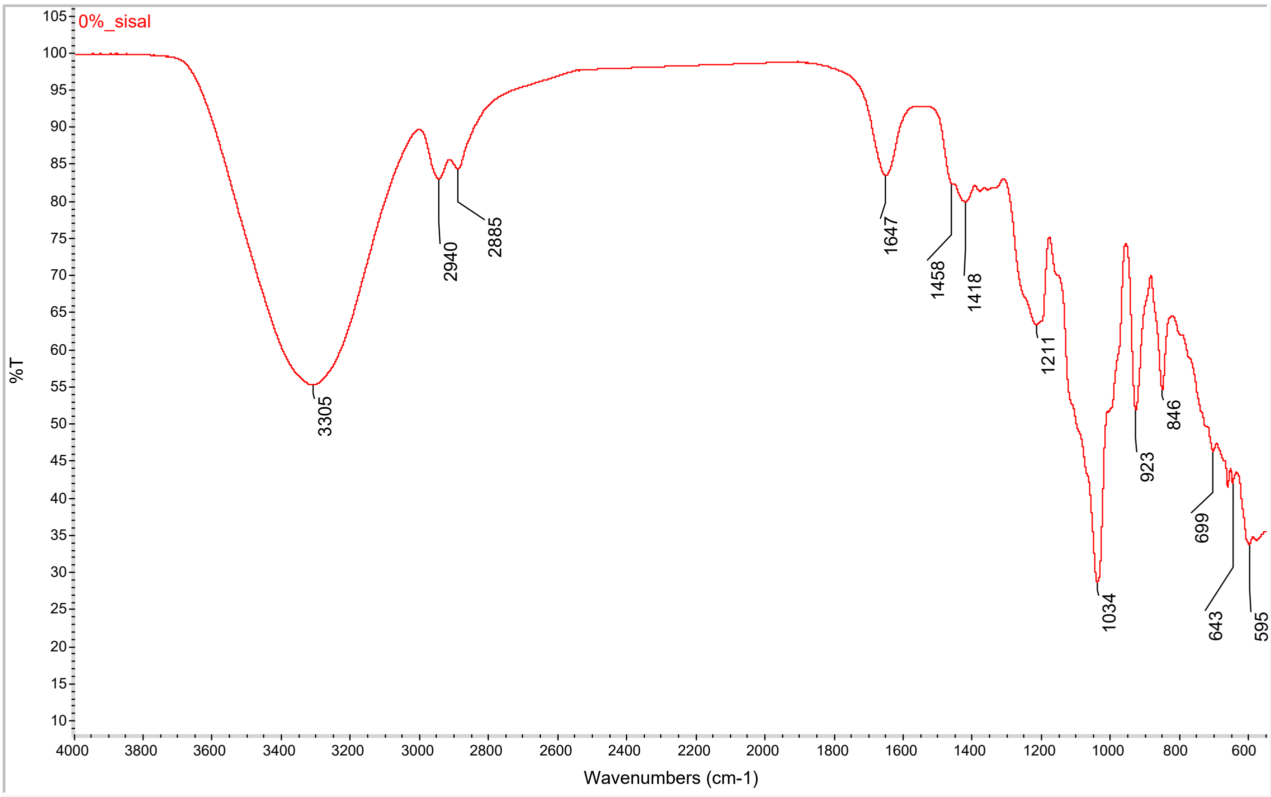

Figure S1: FTIR spectrum of 0% sisal fiber sample

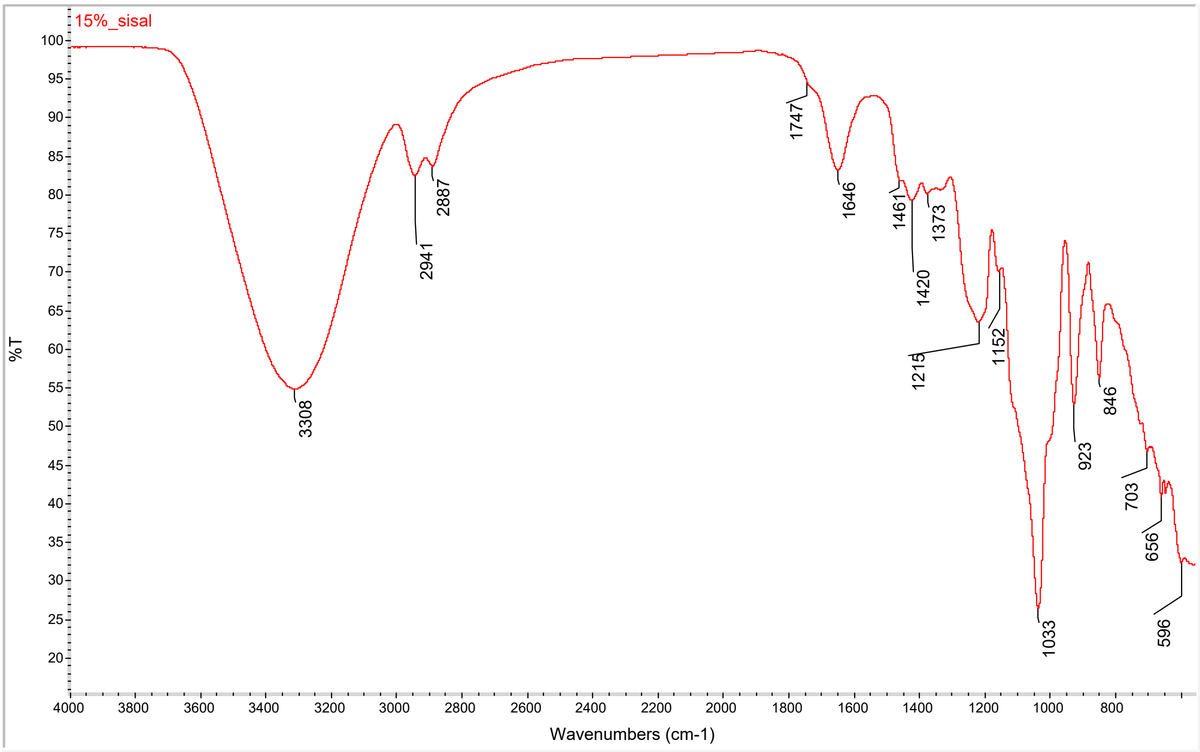

Figure S2. FTIR spectrum of 15 wt% sisal sample

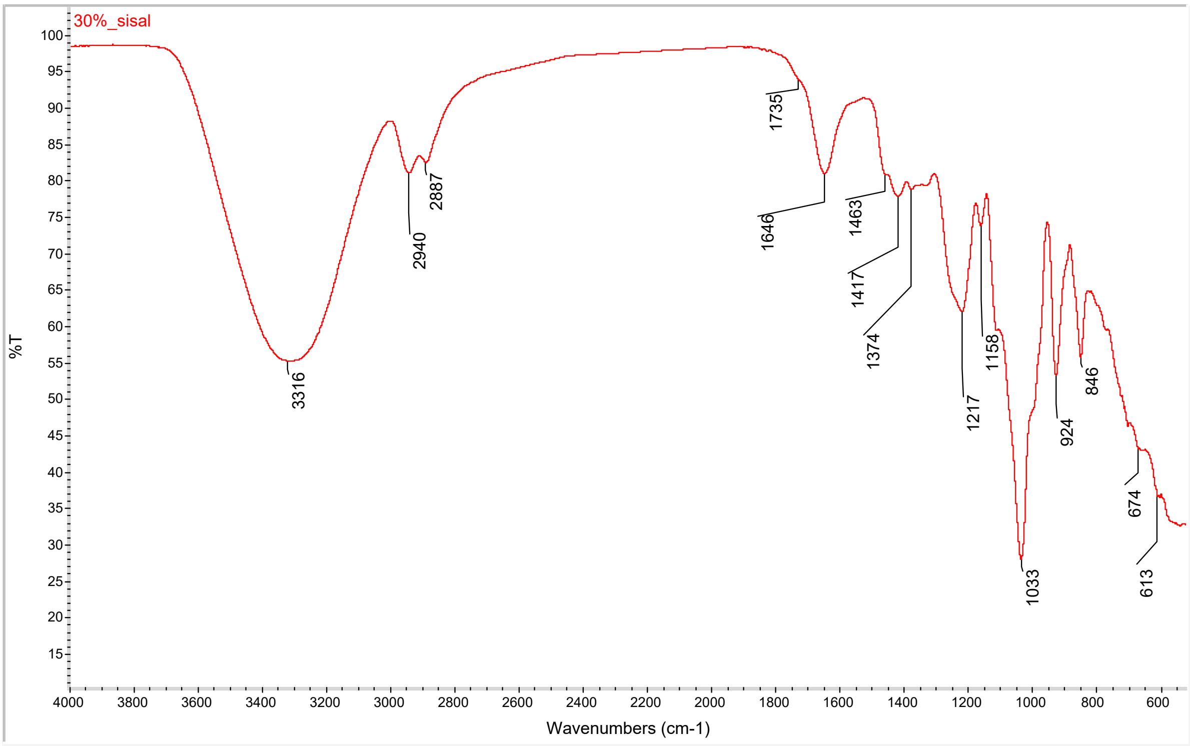

Figure S3: FTIR spectrum of 30% sisal fiber sample

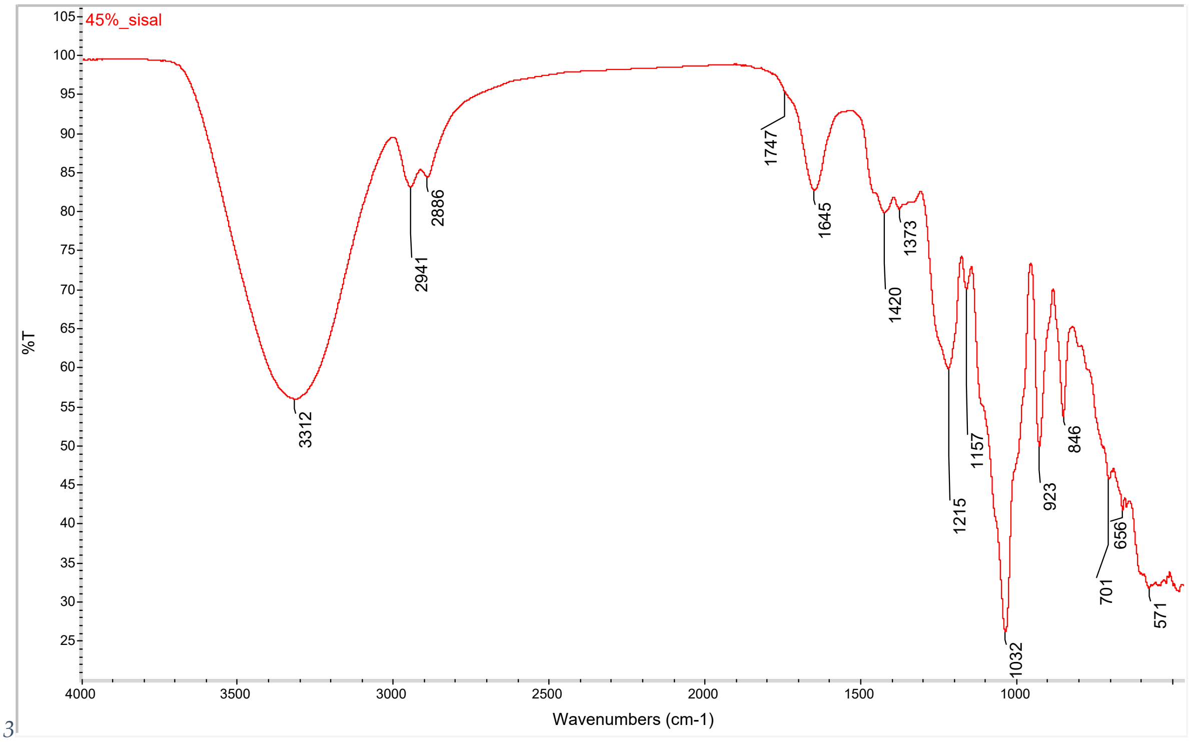

Figure S4: FTIR spectrum of 45% sisal fiber sample

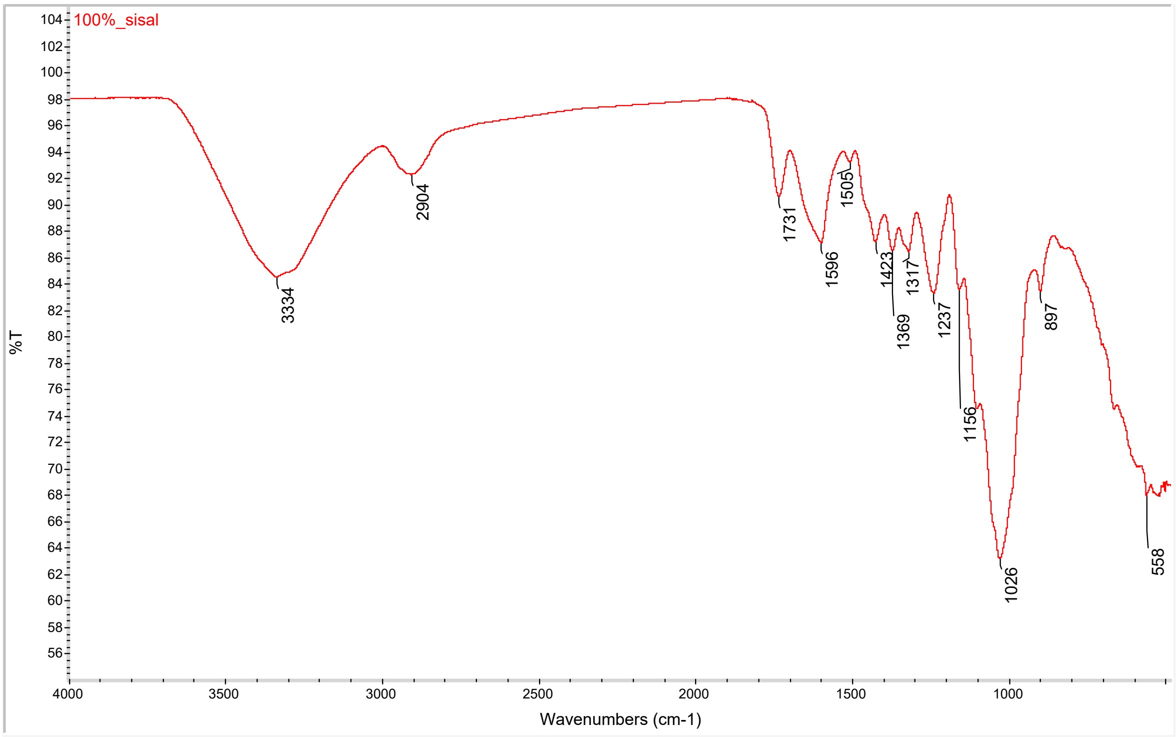

Figure S5: FTIR spectrum of 100% sisal fiber sample

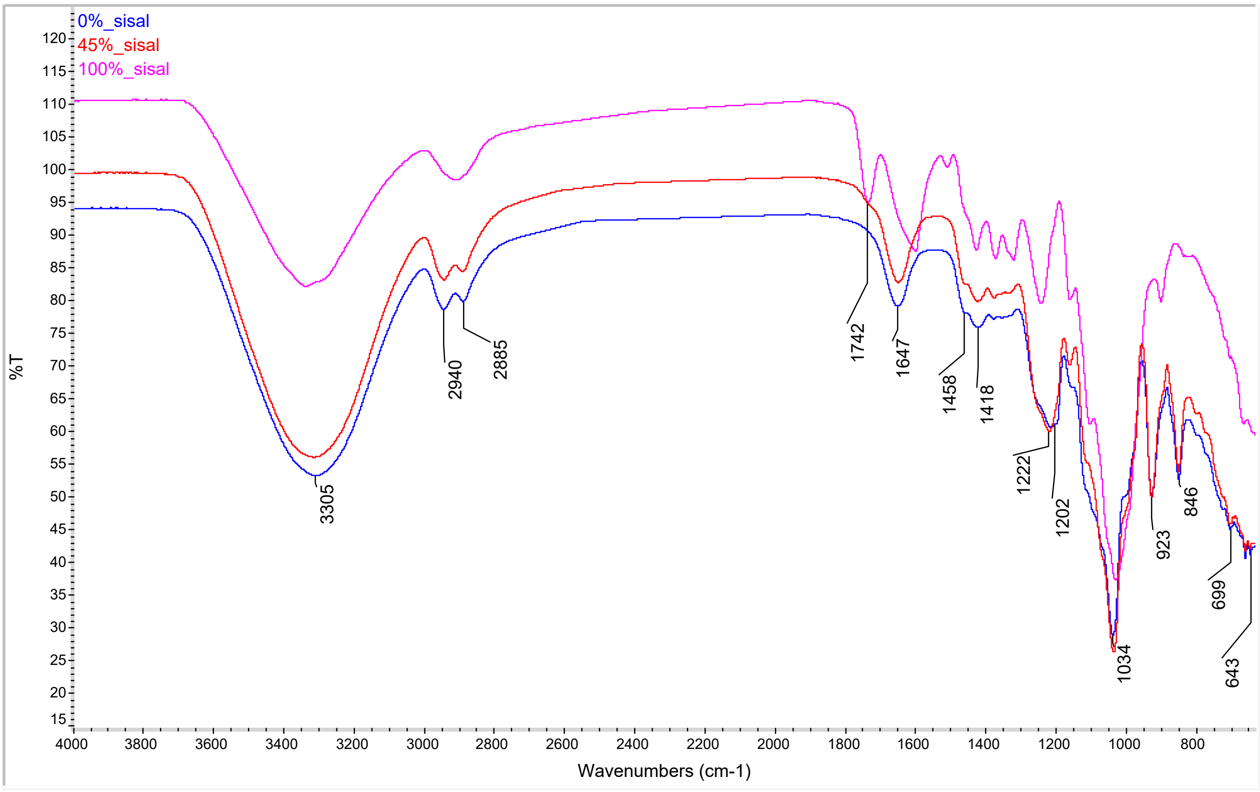

Figure S6. Comparison of 0, 45 and 100% sisal fiber samples

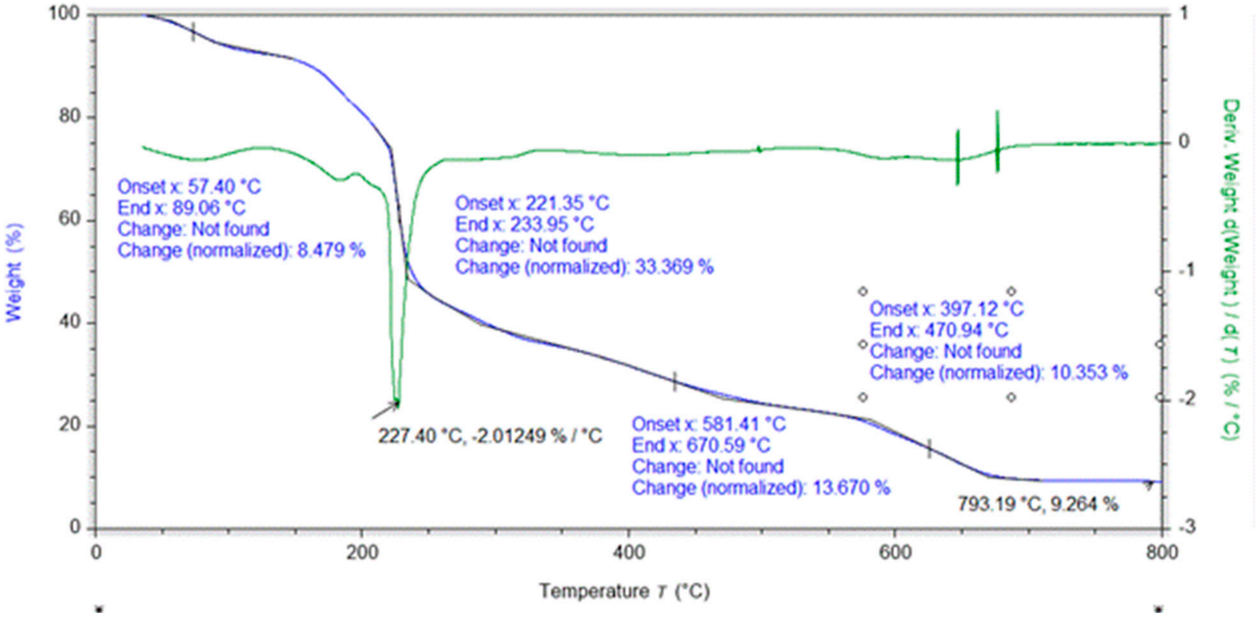

Figure S7. TGA Thermogram and derivative plot of the 0% sisal fiber sample

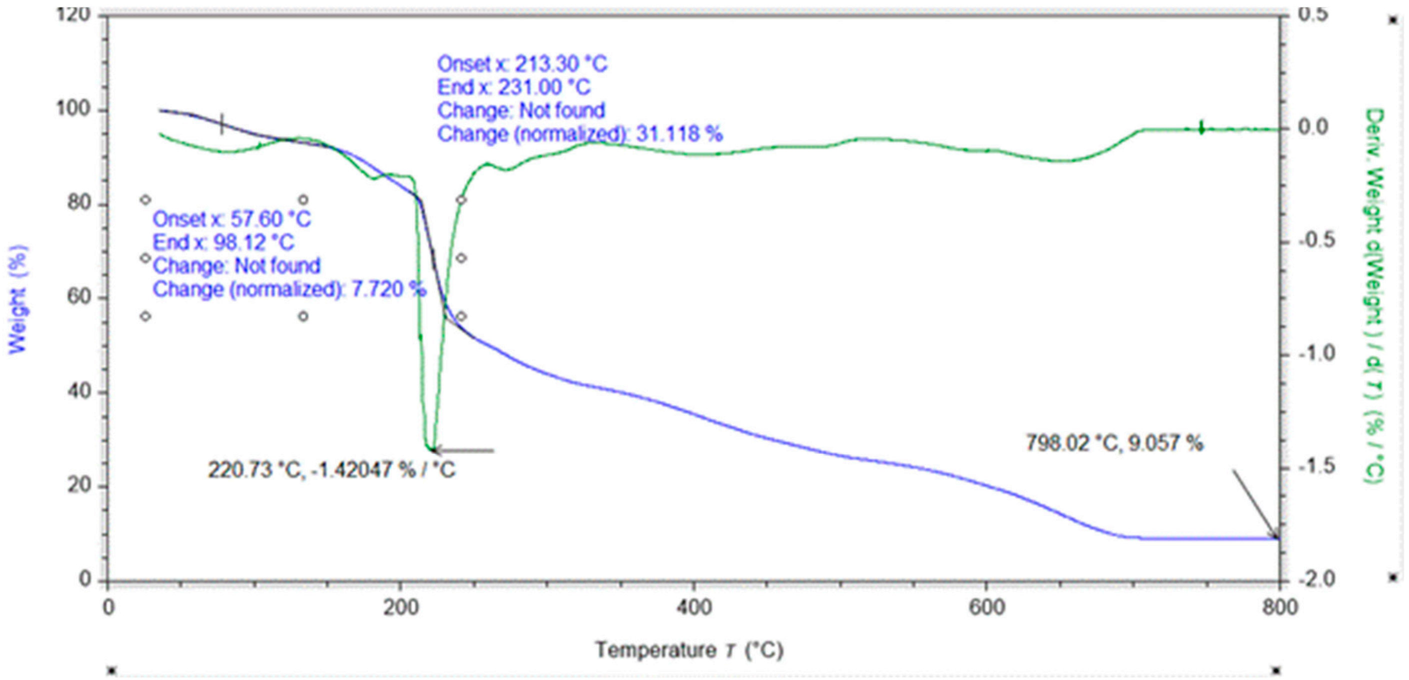

Figure S8: TGA Thermogram and derivative plot of the 15% sisal fiber sample

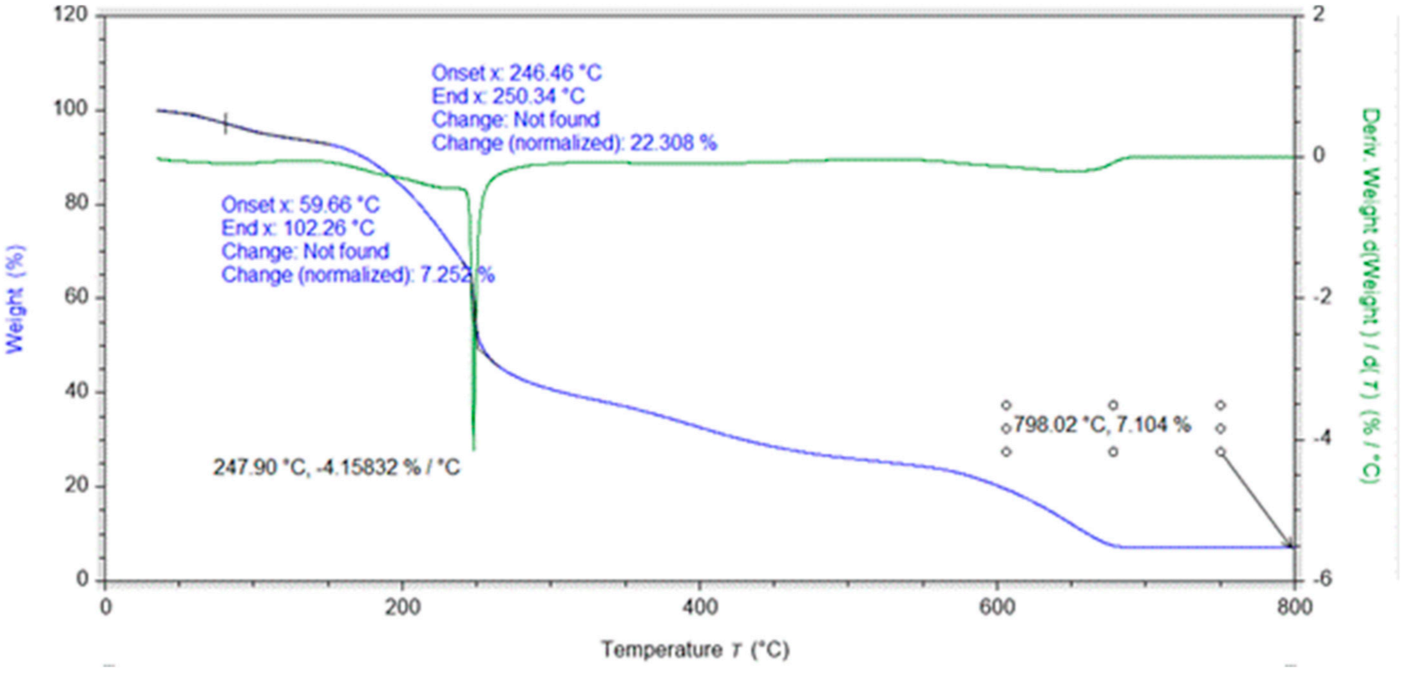

Figure S9: TGA Thermogram and derivative plot of the 30% sisal fiber sample

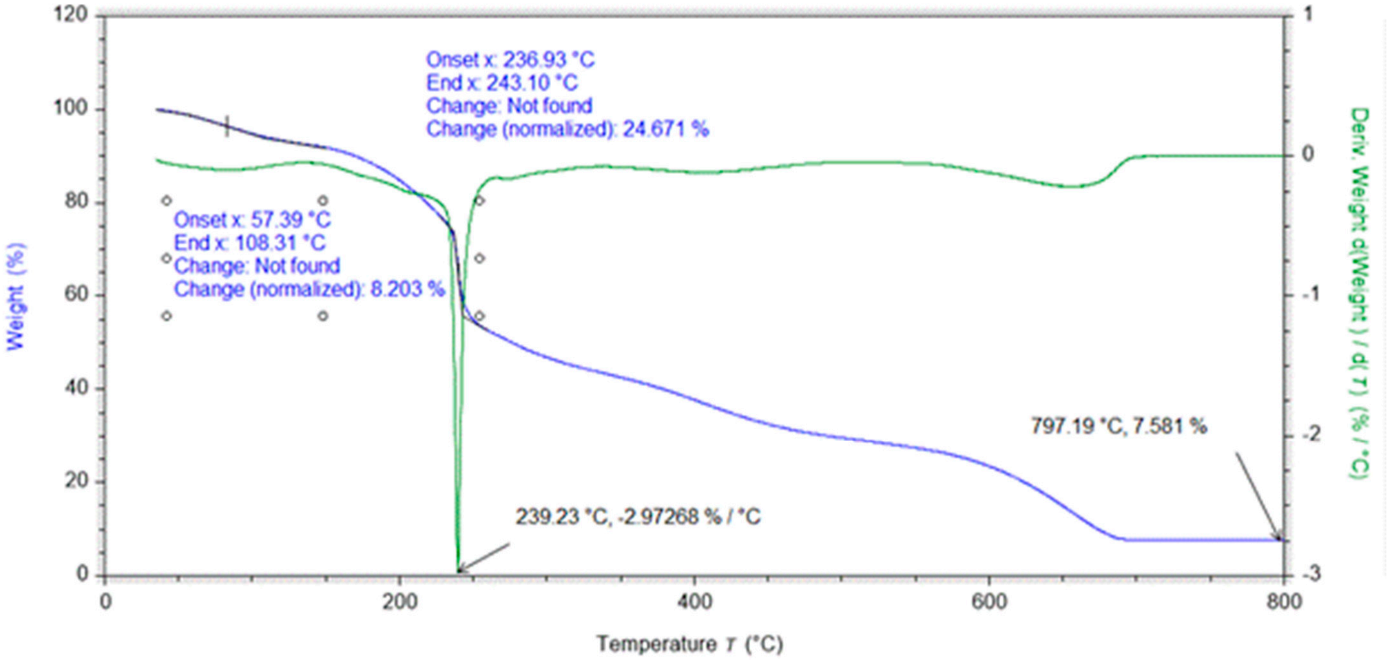

Figure S10: Thermogram and derivative plot of the 45% sisal fiber sample

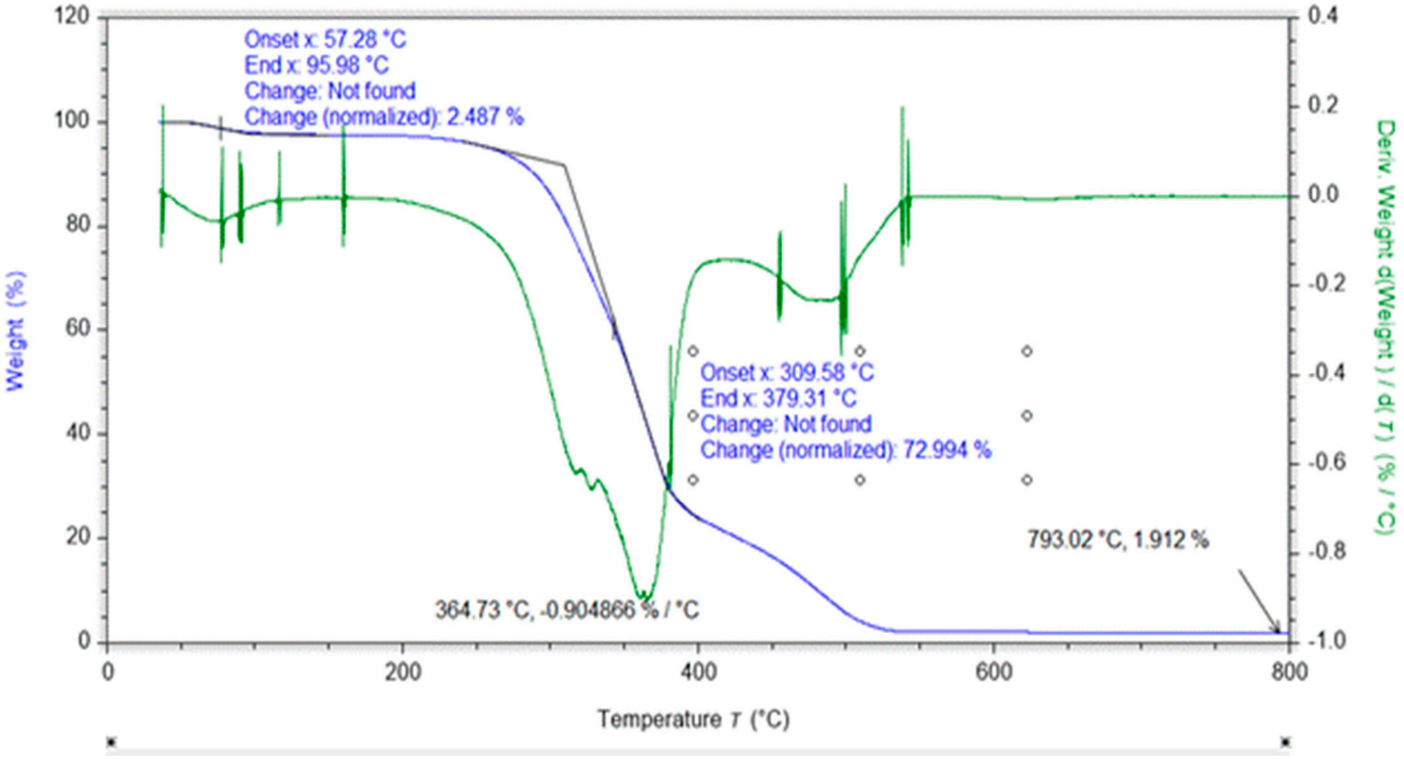

Figure S11: TGA Thermogram and derivative plot of the 100% sisal fiber sample

Table S1: *Mechanical testing specimen dimensions*

Sample	Thickness (mm)	Gauge length (cm)	Width (cm)
0% sisal	0.195	2.23	5
15% sisal	0.249	2.21	5
30% sisal	0.313	2.28	5
45% sisal	0.377	2.28	5

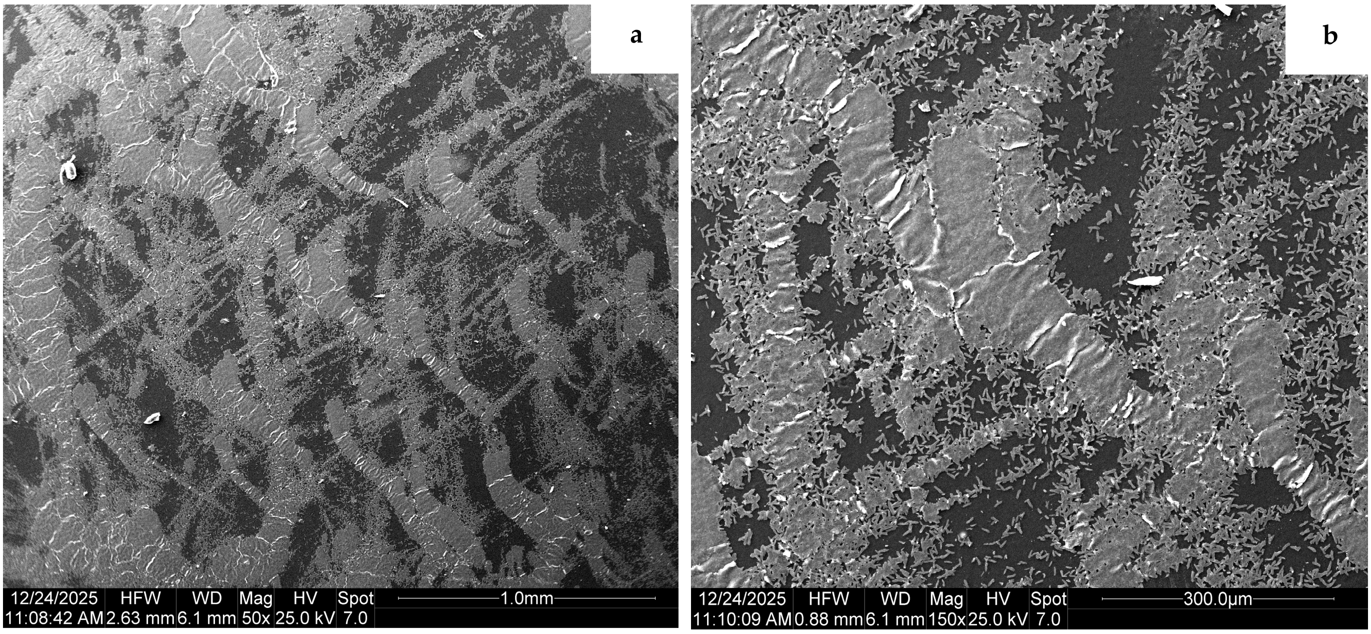

Figure S12: SEM images of RA film a) 50x and b) 150x

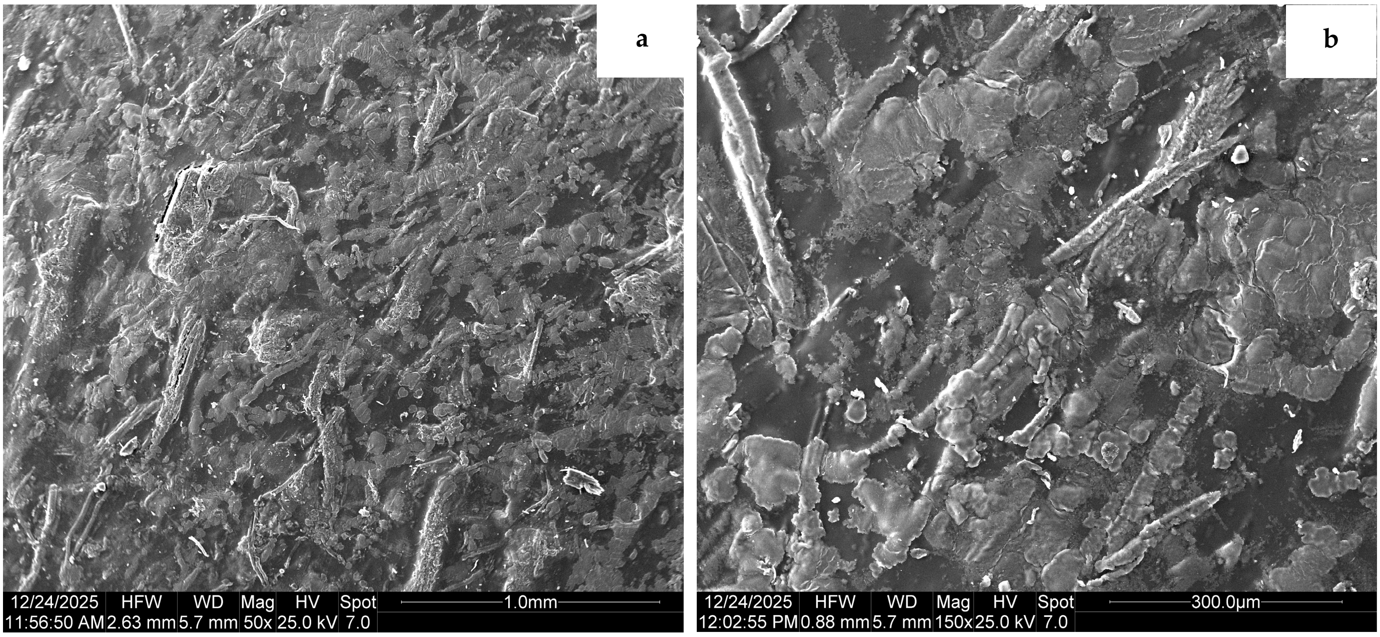

Figure S13: SEM images of 15% sisal film a) 50x and b) 150x

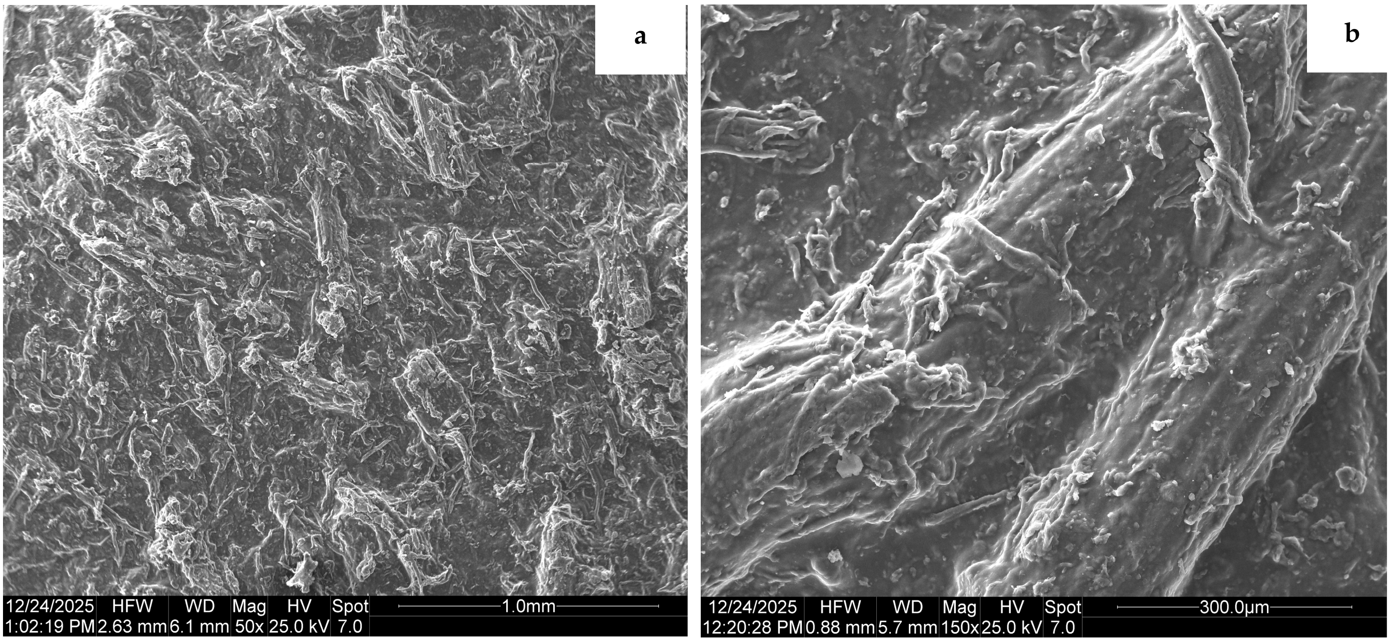

Figure S14: SEM images of 30% sisal film a) 50x and b) 150x

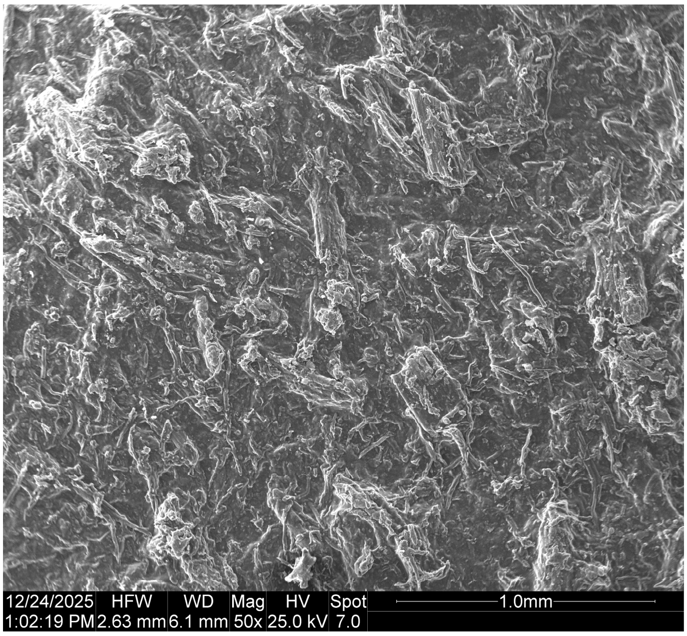

Figure S15: SEM images of 45% sisal film a) 50x and b) 150x
